# Biocontrol Potential of *Androlaelaps casalis* Against Two Key Phytoparasitic Nematodes: *Tylenchulus semipenetrans* and *Meloidogyne incognita*

**DOI:** 10.3390/biology15131013

**Published:** 2026-06-26

**Authors:** Mahmoud M. Al-Azzazy, Suloiman M. Al-Rehiayani

**Affiliations:** Department of Plant Protection, College of Agriculture and Food, Qassim University, P.O. Box 6622, Buraydah 51452, Saudi Arabia; s.alrehiayani@qu.edu.sa

**Keywords:** plant-parasitic nematodes, soil predatory mites, predation rate, survival, life table parameters, biological control

## Abstract

Plant-parasitic nematodes are a significant agricultural challenge, causing extensive damage to most essential crops globally. Predatory soil mites play a significant role as biocontrol agents against plant-parasitic nematodes and other pests in the soil ecosystem. A laboratory trial was conducted to evaluate the potential of the soil-dwelling predatory mite, *Androlaelaps casalis* (Berlese), to suppress the citrus nematode, *Tylenchulus semipenetrans* Cobb, and the root-knot nematode, *Meloidogyne incognita* (Kofoid and White). Our findings show that the predatory mite, *A. casalis,* completed development, oviposition, and survival when fed on second-stage juveniles of both *T. semipenetrans* (*Ts*-J2) and *M. incognita* (*Mi*-J2) and egg mass of *M. incognita* (*Mi*-EM) as prey in the lab in closed arenas at 30 °C, 55% RH. Survivorship was lower on (*Mi*-EM) than on (*Ts*-J2) and (*Mi*-J2). Individuals of *A. casalis* reared on (*Ts*-J2) and (*Mi*-J2) prey demonstrated enhanced performance as compared to (*Mi*-EM) prey. In addition, females laid a total of 48.72, 46.50, and 12.45 eggs on the three types of prey, respectively. Life table parameters showed that feeding of *A. casalis* on (*Ts*-J2) and (*Mi*-J2) led to the greatest intrinsic rate of increase per day (*r_m_* = 0.286 and 0.279 females/female/day), while preying on (*Mi*-EM) offered the lowest reproduction rate (*r_m_* = 0.092). In conclusion, this study provides novel insights into the biology and predatory performance of *A. casalis* under controlled laboratory conditions, providing foundational evidence that may inform the development of future sustainable nematode management strategies.

## Simple Summary

This study examined whether the soil-dwelling predatory mite *Androlaelaps casalis* can feed, develop, and reproduce on two major plant-parasitic nematodes, *T. semipenetrans* and *Meloidogyne incognita*. Under lab conditions, the mite completed its life cycle when feeding on juvenile stages of both nematodes and on *M. incognita* egg masses. However, its performance was weaker on egg masses. Reproduction and population growth were highest when the mite fed on juvenile nematodes, indicating better suitability of these prey types. Overall, the results highlight *A. casalis* as a promising candidate for future biological control strategies targeting harmful nematodes.

## 1. Introduction

Plant-parasitic nematodes represent one of the most destructive threats to global agriculture, consistently driving severe yield losses through root damage, disrupted nutrient acquisition, and marked reductions in plant vigor [1,2]. With the increasing global population and the resultant demand for food security, managing these nematodes is crucial for sustainable agricultural practices [3]. The citrus nematode, *Tylenchulus semipenetrans*, is a serious pest affecting citrus crops worldwide, causing a condition known as “citrus slow decline,” which reduces citrus fruit yield and quality, with yield losses estimated at 15–30% globally [4,5]. This nematode poses a considerable challenge due to its adaptability and wide host range, demanding effective management strategies. In addition, the root-knot nematode, *Meloidogyne incognita*, is an important agricultural pest that affects a wide range of crops, causing substantial yield losses ranging from 10% to 80% across crops [6,7]. Despite chemical nematicides remaining a common control method [8], exploring more sustainable alternatives is required to mitigate pesticide risks posed to humans and the environment [9]. The transition from chemical nematicides to biological control could be strengthened with a more critical discussion of the limitations and adoption barriers of current biocontrol strategies. The biological control agents offer a promising path towards sustainable management of *T. semipenetrans* and *M. incognita*. One promising avenue is the biocontrol of plant-parasitic nematodes using predatory soil mites [10]. Predatory soil mites belong to the Acari subclass and play pivotal roles in the regulation of soil food webs, widely distributed in diverse soil environments [11]. These mites are natural enemies of nematodes and other small soil organisms, preying on their eggs, juveniles, and adults [12,13,14]. Their predatory behavior, ability to coexist with other soil biota, and adaptability to fluctuating environmental conditions make them significant tools in integrated pest management programs [11]. Unlike chemical treatments, predatory mites do not pose risks to human health or the environment and can provide a long-term solution by establishing a natural balance within the soil ecosystem [15].

A substantial body of research demonstrates that numerous soil-dwelling predatory mites possess strong potential for suppressing plant-parasitic nematodes under both laboratory and field conditions [10,15,16,17,18,19,20,21]. Collectively, these predators are capable of markedly reducing nematode populations, thereby mitigating their impact on crop health. For example, Stirling et al. (2017) reported that the soil mite *Protogamasellus mica* (Athias-Henriot) significantly reduced populations of plant-parasitic nematodes, consuming up to 50 individuals of *Pratylenchus zeae* Graham and 40 s-stage juveniles of *Meloidogyne javanica* (Treub) per day [19]. Similarly, the laelapid mite *Hypoaspis calcuttaensis* (Bilgrami) has been shown to exhibit a strong predatory capacity against both saprophagous and plant-parasitic nematodes [22].

Likewise, the oribatid mite, *Pergalumna* sp., has been confirmed to prey on 42 adults and J2 juveniles of *Pratylenchus coffeae* (Zimmermann) and 18 J2 juveniles of *M. javanica* per day. Ref. [23] assessed that the predacious mite *Blattisocius dolichus* Ma greatly decreased the density of the burrowing nematode, *Radopholus similis* (Cobb). Al-Azzazy and Al-Rehiayani (2022) reported that the cunaxid mite *Cunaxa capreolus* (Berlese) (Acari: Cunaxidae) fed on (*Mi*-EM), (*Mi*-J2), and (*Ts*-J2) as food sources under lab conditions [10]. However, to the best of our knowledge, no study has evaluated the effectiveness of the soil-dwelling predatory mite *Androlaelaps casalis* against plant-parasitic nematodes, despite its documented success in preying on free-living nematodes.

The family Laelapidae currently comprises more than 1088 described species distributed across 73 genera worldwide [16]. Most species of this family are free-living predators fed on small soil-inhabiting organisms. Knowledge of the Laelapidae fauna in the Kingdom of Saudi Arabia remains limited, with only five species documented to date: *Androlaelaps casalis* (Berlese), *Cosmolaelaps simplex* (Berlese), *Hypoaspis zaheri*, *Hypoaspis dactylifera*, and *Cosmolaelaps qassimensis*. These species have been reported in the debris of date palm trees, the root zones of citrus trees, and eggplant fields in the Qassim region [24]. The soil-dwelling predatory mite *Androlaelaps casalis* (Berlese) (Acari: Laelapidae) is a cosmopolitan, free-living species inhabiting diverse microhabitats and capable of exploiting a broad range of food resources. Its diet includes dried blood, farinaceous materials, eggs of various mites [25], the flour mite *Acarus siro* Linnaeus and the mycophagous mite *Tyrophagus putrescentiae* (Schrank) [26], several stored-product mite pests and inert substrates such as brewer’s yeast [27], immature stages of the poultry red mite *Dermanyssus gallinae* (De Geer) [28], free-living nematodes [29], and beetle larvae [30]. *Androlaelaps casalis* is also among the most abundant predatory mites in poultry litter and related habitats [31,32]. Notably, the commercially available strain of *A. casalis* differs from field populations, belonging to a distinct mitochondrial haplotype (16S rRNA) and originating from mites collected in bird nests [32].

Given the divergent and poorly resolved ancestries within this taxon, its feeding ecology remains insufficiently understood. However, this species is presently approved for use as a biocontrol agent against some exogenous mite populations in France [32]. Given the lack of prior evidence regarding this predator’s ability to suppress plant-parasitic nematodes, we hypothesize that *A. casalis* will display favorable life-history traits, robust predatory activity, and sustained population growth when feeding on *T. semipenetrans* and *M. incognita*. Confirmation of these responses under field conditions would substantiate the potential of *A. casalis* as an effective biological control agent against economically important nematodes.

## 2. Materials and Methods

### 2.1. Nematodes

The citrus nematode, *T. semipenetrans,* and the root knot nematode, *M. incognita*, were obtained from an untreated orange orchard and tomato greenhouse, respectively, at the farm of the College of Agriculture and Food (26.292385° N, 43.792975° E), Qassim Region, Saudi Arabia. Egg masses of *M. incognita* for these trials were acquired from an untreated tomato greenhouse (*Solanum lycopersicum* L. cv. Pritchard). Roots of tomato containing egg masses of *M. incognita* were collected in a Pyrex glass tray and cleaned free of dirt and soil using purified water. The tomato roots were carefully sectioned into fragments measuring 1.5–2.5 cm in length, each containing three egg masses, and subsequently provided to *A. casalis* as prey. J2 juveniles of *T. semipenetrans* were obtained from soil blended with orange roots, while *M. incognita* were obtained from tomato roots. Nematode juveniles were extracted using a combination of sieving followed by sucrose centrifugation [33]. The sieve used had a pore size of 39 micrometers. Sieve size: 400-mesh, equivalent to a 39-micrometer opening. Using a 40× magnification dissecting microscope, one milliliter of nematode solution was transferred to a nematode counting slide, and the juveniles’ numbers were set to 150 J2/mL for the experiments. Among the most effective techniques for isolating both active and slow-moving, inactive nematodes is centrifugal flotation, which was employed in this investigation [34].

### 2.2. Predatory Mite

The *Androlaelaps casalis* used in these trials was obtained from untreated orange orchard soil and tomato greenhouse soil at the Research Station, Al-Qassim Province, Saudi Arabia. Soil samples were brought to the lab of Acarology, College of Agriculture and Food, Qassim University. The predatory mite *A. casalis* was extracted using the Berlese–Tullgren technique with funnels measuring 25 cm in diameter (Krantz, 1978) [35]. The light source controlled by a rheostat for one day, by which time > 95% of the active predacious mites were removed and kept in a dark environment at 30 ± 1 °C, 55 ± 5% RH, with (*Mi*-J2) provided as a food source. The specimens were identified as *A. casalis* based on adult female morphology. The mites were cleared in lactic acid and mounted in Hoyer’s medium for examination under a compound microscope equipped with phase contrast. Identification followed the diagnostic characteristics and species keys of Krantz (1978) [35]. Confirmation was based on the shape and ornamentation of the dorsal and ventral shields, the chaetotaxy of the dorsal shield and legs, the configuration of the spermatheca, and the characteristic cheliceral pilus dentilis with an inflated base. All the examined specimens conformed to the diagnostic combination of characters defining *A. casalis*.

### 2.3. Experimental Arenas

All the experiments were carried out in rearing arenas with dimensions of 2.5 cm in diameter and 1.2 cm in depth. The arenas were filled with a 1:7 mixture of plaster of Paris and activated charcoal down to a depth of 0.5 cm. The arenas were covered with a glass slide, and the two sections were fastened together with a binder clip to prevent the mites from escaping.

### 2.4. Prey

Three different types of prey were assessed for their influence on development, survival rate, longevity, predation rate, fecundity, and life table parameters of *A. casalis*.

1. J2 juveniles of citrus nematode, *T. semipenetrans* (*Ts*-J2);
2. J2 juveniles of root-knot nematode, *M. incognita* (*Mi*-J2);
3. Egg mass of root-knot nematode, *M. incognita* (*Mi*-EM).

### 2.5. Feeding Studies of A. casalis on (Ts-J2), (Mi-J2), and (Mi-EM)

To obtain same-aged cohorts of predator eggs for the different biological experiments, gravid females of *A. casalis* were transferred into rearing arenas using a damp brush, with J2 juveniles of citrus nematode, *T. semipenetrans* as prey, and permitted to lay eggs for two days, and the resulting eggs were subsequently separated for the various biological trials. Each egg was transferred to its designated rearing arena using a fine brush, and the newly emerged larvae (40 per trial) were provided with one of the three prey types under evaluation. The number of replicates was 40 per diet. Within 24 h of emergence, newly emerged female adults were allowed to mate with an active male from the stock culture. Then, the males were transferred to different arenas and raised separately until their death. Three trials were designed to quantify predation on (*Ts*-J2), (*Mi*-J2), and (*Mi*-EM). In the first trial, 100 (*Ts*-J2) were added daily to each rearing arena.

In the second trial, 100 (*Mi*-J2) were added daily to each rearing arena. In the third experiment, three (*Mi*-EM) and distilled water droplets were added to each rearing arena every day, and each rearing arena was closed and placed in an incubator at 30 ± 1 °C, 55 ± 5% RH in a dark environment. For every trial, the number of nematodes devoured or otherwise destroyed by *A. casalis*/day was recorded. The number of J2 consumed was calculated as the difference between the initial and final counts. For egg masses, the remaining intact eggs were counted and compared with the initial egg number to estimate the number of eggs consumed. J2 or eggs were classified as “eaten” or “destroyed” when they showed clear signs of predation, such as a ruptured or collapsed cuticle or disintegrated internal contents, whereas individuals or eggs with intact, turgid bodies and undisturbed contents were recorded as “uneaten”. To maintain a steady supply of prey, the predators were moved to new arenas every two to three days, and the prey was replaced every day. Developmental, survival, predation, fecundity, and behavior observations were recorded twice daily. The eggs of the predator and residual prey material were extracted from the rearing arenas daily. The survival to the adult stage and developmental time of the progeny (N = 40) from each female in each treatment were recorded to calculate life-table parameters, according to Hulting et al. (1990) [36]. The female survival rate for each prey was determined, accounting for deaths that occurred in the egg or other immature stages. A dissecting microscope (Olympus Optical, Tokyo, Japan) was used to make the observations.

### 2.6. Statistical Analysis

Life-history parameters, including survival rate, number of prey consumed, and egg production of all *A. casalis* individuals on the three prey types, were analyzed using simple correlation, the SAS 9.4 statistical package [37], and analysis of variance (ANOVA). Differences among means were separated using Tukey’s honest significant difference (HSD) test.

## 3. Results

### 3.1. Development Time, Survival Rate, and Life History

The results show that *A. casalis* could normally reach the adult stage and successfully preyed on (*Ts*-J2), (*Mi*-J2), and (*Mi*-EM). The total development times for *A. casalis* females and males were significantly influenced by prey species. The developmental time of *A. casalis* on the tested prey is shown in Table 1 and Table 2. The total development time of *A. casalis* was slightly quicker in males than in females. There was no significant difference between the (*Ts*-J2) and (*Mi*-J2) with respect to the period of development of the predatory mite. Comparing (*Ts*-J2), (*Mi*-J2), and (*Mi*-EM) as food sources, the *A. casalis* population developed faster for males and females when feeding on (*Ts*-J2) and (*Mi*-J2) than when feeding on (*Mi*-EM). The survival rates showed significant differences occurring in the percentage of mites reaching maturity on the three types of prey (F = 1.4; df = 2, 7; *p* = 0.0031), which were 96.80 ± 1.46, 94.50 ± 1.28, and 66.00 ± 2.30 for *A. casalis* fed on (*Ts*-J2), (*Mi*-J2) and (*Mi*-EM), respectively (Table 3). The prey type had a clear, significant impact on adult longevity (F = 41.7; df = 2, 6; *p* < 0.001 for females; F = 24,7; df = 2, 45; -*p* < 0.001 for males; Table 2). Compared to (*Mi*-EM), *A. casalis* females and males lived longer when fed the (*Ts*-J2) and (*Mi*-J2) diet.

### 3.2. Fecundity

The pre-oviposition period of *A. casalis* reared on (*Ts*-J2) and (*Mi*-J2) was significantly shorter than mites fed (*Mi*-EM) (F = 2.68; df = 3, 5; *p* = 0.004; Table 2). Additionally, a longer oviposition period was observed when females were reared on (*Ts*-J2) and (*Mi*-J2). In contrast, it was the quickest when *A. casalis* was fed on (*Mi*-EM) (Table 2), but a longer post-oviposition period was observed when females were reared on (*Mi*-EM). The fecundity was significantly lower when the mites were fed (*Mi*-EM) (12.45 eggs/female), while the predatory mites fed on (*Ts*-J2) and (*Mi*-J2) produced a significantly greater number of eggs (48.72 and 46.50 eggs/female, respectively) (F = 3.14; df = 3, 92; *p* = 0.002; Table 4).

### 3.3. Predation Rate

The findings demonstrate that *A. casalis* larvae fed on the tested prey species, and predation activity started just after hatching. (*Ts*-J2) was the most suitable prey type for *A. casalis* compared with (*Mi*-J2) and (*Mi*-EM). The age-stage-specific predation rate was multiplied by increasing the stage of the predatory mite, *A. casalis*, for the three types of prey; therefore, compared to the immature stages, the adults consumed more prey. Table 5 shows the numbers of (*Ts*-J2), (*Mi*-J2), and (*Mi*-EM) prey devoured by predatory males and females. The average daily predation rate was 79.14, 73.69, and 2.48 prey for female immature stages (*p* = 0.0014), and 73.09, 68.44, and 2.18 prey for male immature stages (*p* = 0.0019) on (*Ts*-J2), (*Mi*-J2), and (*Mi*-EM), throughout the juvenile stages, respectively. The predation rate increased from pre-oviposition to oviposition periods and decreased in the post-oviposition phase. Subsequently, daily predation reduced with age. The maximum consumption rate was recorded during the oviposition period, with the female consuming an average of 109.83 (*Ts*-J2), 97.09 (*Mi*-J2), and 4.49 (*Mi*-EM), respectively. Compared to males, females devoured a significantly greater quantity of prey through the adult stage and the entire lifespan (F = 4.21; df = 3, 23; *p* = 0.001; Table 5). The results show that the mean number of prey consumed on diets of (*Ts*-J2), (*Mi*-J2), and (*Mi*-EM) by the average individual *A. casalis* female during its lifespan was 4432.09, 3827.12, and 87.17 prey, respectively.

### 3.4. Life Table Parameters

The life table parameters demonstrated that the prey species had significant effects on *A. casalis* life history (Table 6). The females reared on (*Ts*-J2) and (*Mi*-J2) exhibited the greatest net reproductive rate (*R_o_*), intrinsic rate of increase (*r_m_*), and finite rate of increase (λ). On the other side, *A. casalis* individuals reared on (*Ts*-J2) and (*Mi*-J2) had the shortest mean generation time. The life table parameters were calculated as the net reproduction rate (*R_o_*) of 30.19, 28.49, 8.79; the mean generation time (*T*) of 24.37, 23.96, 31.52 d; the intrinsic rate of population increase (*r_m_*) 0.286, 0.279, 0.092 day^–1^; and the finite rate of increase (λ) of 1.522, 1.498, 0.617 for *A. casalis* when fed on (*Ts*-J2), (*Mi*-J2) and (*Mi*-EM), respectively. In addition, *A. casalis* fed on (*Mi*-EM) had the longest mean generation time and the minimal values of life table parameters.

## 4. Discussion

This investigation represents the first documented evaluation of the biology and predatory capacity of laelapid mite, *A. casalis*, when offered the citrus nematode, *T. semipenetrans*, and the root-knot nematode, *M. incognita,* as prey. The results indicate that both prey species constitute highly nutritious and suitable food resources, promoting accelerated developmental rates and markedly enhanced fecundity in the predator [38]. This is an important step in improving management strategies against the citrus nematode, *T. semipenetrans,* and the root-knot nematode, *M. incognita*. In this research, the prey species had a noticeable impact on immature *A. casalis*’ development. When *A. casalis* fed on (*Ts*-J2) and (*Mi*-J2), there was no significant difference in their life-history parameters, and they could develop and complete their life cycles. Additionally, *A. casalis* had higher fertility and a shorter mean generation time when feeding on (*Ts*-J2) and (*Mi*-J2) than when feeding on (*Mi*-EM), which suggests that (*Ts*-J2) and (*Mi*-J2) are more suitable for the development and reproduction of the predatory mite, *A. casalis*. The total development time of *A. casalis* females fed (*Ts*-J2) (at 30 °C and 55% RH, 5.46 days) in the present research was shorter than for *A. casalis* fed on the brewer’s yeast and storage mite, *Glycyphagus domesticus* (De Geer) at 30 °C, 6.28 days [27]. Contrastingly, the development of *A. casalis* immature females was longer in our research (5.46 d) than reported when fed on *Tyrophagus putrescentiae* (Schrank), 5.00 days [29]. The developmental time of immature females of *A. casalis* fed on free-living nematodes at 30 °C, 70% R.H was 5.50 days, which was very close to the present findings against (*Ts*-J2) [29].

The maximum fecundity was attained when *A. casalis* fed on (*Ts*-J2) (48.72 eggs/female). This amount was greater in comparison to mites that fed on the acarid mite, *T. putrescentiae* (31.8 eggs/female), and on free-living nematodes (40.0 eggs/female) [29]. These results suggest that the diet of (*Ts*-J2) and (*Mi*-J2) is more appropriate for *A. casalis* reproduction. In the present study, prey species also affected the adult longevity of *A. casalis*; both females and males had longer lifespans when fed (*Ts*-J2) and (*Mi*-J2). The female lifespan in the present study (46.10 ± 2.14 days) was close to that of females feeding on free-living nematodes (44.0 ± 0.7) at 25 °C [29]. The reproductive potential of predatory mites also plays a crucial role in their efficacy as biocontrol agents. Under favorable conditions, predatory mites can reproduce rapidly, which allows them to maintain high population densities and provide continuous suppression of nematode populations [14]. In addition, the high fertility of predatory mites enables them to quickly establish and control nematode populations, especially when prey is abundant [33]. Our laboratory studies clearly demonstrate that the predatory mite, *A. casalis*, can destroy or damage a significant number of *T. semipenetrans* and *M. incognita*. Al-Azzazy and Al-Rehiayani (2022) previously discovered that the Cunaxid mite, *C. capreolus,* consumed an average of 36.78 J2 juveniles of *T. semipenetrans* and 47.57 J2 juveniles of *M. incognita* per day [10]. Still, the level of predation rate in the current trials appeared to be significantly higher, which showed that *A. casalis* possesses a high predatory capacity on *T. semipenetrans* and *M. incognita*. The life table parameters of *A. casalis* were influenced by prey species. The population growth parameters were more favorable for *A. casalis* fed on (*Ts*-J2) and (*Mi*-J2) compared to (*Mi*-EM). This is verified by the intrinsic rate of increase (*r_m_*), which was 0.286 and 0.279 on (*Ts*-J2) and (*Mi*-J2), while it was 0.092 on (*Mi*-EM). Undoubtedly, the observed low potential is not an inherent quality of *A. casalis* but rather the result of the unsuitability of the (*Mi*-EM) food source. This assertion demonstrates the high fecundity of *A. casalis* when fed on (*Ts*-J2) and (*Mi*-J2). Likewise, it was previously discovered that *C. capreolus* fed and reproduced when it was provided with (*Ts*-J2) and (*Mi*-J2). Similarly, it was observed that its reproductive potential was lower when feeding on (*Mi*-EM) than on nematodes [10].

Several biological investigations have confirmed that superior food sources yielded higher values for life table parameters [39,40,41]. According to Orion et al. (2001), the gelatinous matrix of (*Mi*-EM) may act as an obstacle to preventing the invasion of certain small arthropods and soil-dwelling predatory mites [42]. In a previous study, the predatory mite, *A. casalis*, showed high predation levels on the poultry red mite, *D. gallinae*, under lab conditions. It favored *D. gallinae* over astigmatic mites [30]. Furthermore, the abundances of *A. casalis* contrasted with those of *D. gallinae* in farm constructions, suggesting trophic associations between the predator population and *D. gallinae* in the field [43]. These results suggest that *A. casalis* is a promising control agent for the citrus nematode, *T. semipenetrans*, and the root-knot nematode, *M. incognita*, and it is hoped that the findings reported here will stimulate additional work on mite predators of nematodes.

### Limitations and Future Directions

Although the current study provides detailed insights into the biological performance of the soil-dwelling predatory mite, *A. casalis*, under lab conditions, various factors may limit the direct translation of these results to field environments. Laboratory assays are conducted under constant prey availability, temperature, and humidity, whereas field conditions are characterized by fluctuating nematode densities, complex soil–ecological interactions, and substantial microclimatic variability. Such factors may influence predator foraging efficiency, survival, and reproductive output in ways not captured under controlled conditions. Furthermore, the spatial heterogeneity of the soil structure and the presence of competing or interfering soil fauna may alter predator–prey encounter rates. Future research should therefore include semi-field and field-scale evaluations to assess predator establishment, persistence, and efficacy under natural environmental fluctuations. Integrating these studies with optimal application timing, compatibility with existing pest management practices, and assessments of predator release strategies will be essential for determining the practical feasibility of deploying *A. casalis* as a biological control agent in agricultural systems.

## 5. Conclusions

This study provides novel insights into the biology and predatory performance of *Androlaelaps casalis* under laboratory conditions and may inform future sustainable nematode management strategies. Our findings indicate that the predatory mite *A. casalis* can successfully develop and reproduce when fed the citrus nematode, *T. semipenetrans*, the root-knot nematode, *M. incognita*, and egg masses of *M. incognita*. Owing to its strong predatory capacity, *A. casalis* shows promise as a biological control agent for *T. semipenetrans* and *M. incognita*. Despite the substantial efficacy observed in laboratory settings, its performance in field conditions requires further verification. In addition, future studies can further optimize the production process of *A. casalis* and explore its application effects in different crops and environments to promote its widespread use in the agricultural field.

## Figures and Tables

**Table 1:** Mean (±SE) immature development days of *Androlaelaps casalis* fed on J2 juveniles of *T. semipenetrans*, J2 juveniles of *M. incognita*, and egg mass of *M. incognita* at 30 ± 1 °C, 55 ± 5% RH.

Biological Parameters	Prey Species			
	Predator Gender	J2 Juveniles of *T. semipenetrans* *n* = 40	J2 Juveniles of *M. incognita* *n* = 40	Egg Mass of *M. incognita* *n* = 40
Egg incubation period	Female	2.16 ± 0.09 a	2.20 ± 0.10 a	2.50 ± 0.18 b
	Male	2.05 ± 0.06 a	2.00 ± 0.14 a	2.40 ± 0.16 b
Larva	Female	1.00 ± 0.08 a	1.00 ± 0.10 a	1.35 ± 0.08 b
	Male	1.00 ± 0.06 a	1.00 ± 0.10 a	1.25 ± 0.12 b
Protonymph	Female	1.10 ± 0.07 a	1.05 ± 0.12 a	2.44 ± 0.10 b
	Male	1.05 ± 0.09 a	1.00 ± 0.08 a	2.30 ± 0.12 b
Deutonymph	Female	1.20 ± 0.10 a	1.40 ± 0.08 a	2.45 ± 0.18 b
	Male	1.00 ± 0.08 a	1.35 ± 0.10 a	2.40 ± 0.18 b
Total	Female	5.46 ± 0.24 a	5.65 ± 0.20 a	8.74 ± 0.25 b
	Male	5.10 ± 0.26 a	5.35 ± 0.22 a	8.35 ± 0.25 b

The same letters in lines denote not significantly different using the Tukey test at 5% significance level.

**Table 2:** Mean (±SE) pre-oviposition, oviposition, longevity, post-oviposition, and life span (days) of *Androlaelaps casalis* fed on J2 juveniles of *T. semipenetrans*, J2 juveniles of *M. incognita*, and egg mass of *M. incognita* at 30 ± 1 °C, 55 ± 5% RH.

Biological Parameters	Prey Species		
	J2 Juveniles of *T. semipenetrans* *n* = 40	J2 Juveniles of *M. incognita* *n* = 40	Egg Mass of *M. incognita* *n* = 40
Pre-oviposition period	1.28 ± 0.16 a	1.35 ± 0.22 a	3.48 ± 0.33 b
Oviposition period	35.48 ± 2.12 a	34.15 ± 2.05 a	17.87 ± 3.02 b
Post-oviposition period	4.76 ± 0.61 a	4.95 ± 0.28 a	8.91 ± 0.1.46 b
Female longevity	41.52 ± 2.36 a	40.45 ± 2.75 a	30.26 ± 3.52 b
Male longevity	39.55 ± 2.89 a	38.75 ± 2.36 a	27.64 ± 2.58 b
Female life span	46.98 ± 2.50 a	46.10 ± 2.14 a	39.00 ± 3.28 b
Male life span	44.65 ± 2.35 a	44.10 ± 2.45 a	35.99 ± 3.84 b

The same letters in rows denote not significantly different using the Tukey test at 5% significance level.

**Table 3:** Survival percentages of *Androlaelaps casalis* immature stages fed on J2 juveniles of *T. semipenetrans*, J2 juveniles of *M. incognita*, and egg mass of *M. incognita* at 30 ± 1 °C, 55 ± 5% RH.

Prey Species	Stage-Specific Survival (% ± SE)				Survival to Adulthood (% ± SE)
	Egg	Larva	Protonymph	Deutonymph	
J2 juveniles of *T. semipenetrans* *n* = 40	100 ± 0.00 a	99.62 ± 0.41 a	98.45 ± 1.08 a	97.72 ± 0.30 a	96.80 ± 1.46 a
J2 juveniles of *M. incognita* *n* = 40	100 ± 0.00 a	98.50 ± 0.64 a	96.70 ± 1.12 a	95.11 ± 0.63 a	94.50 ± 1.28 a
Egg mass of *M. incognita* *n* = 40	100 ± 0.00 a	71.90 ± 2.39 b	68.00 ± 2.55 b	67.25 ± 2.84 b	66.00 ± 2.30 b

The Tukey HSD test indicates that there is no significant difference between means that are followed by the same letter inside each column (*p* < 0.05).

**Table 4:** Fecundity of *Androlaelaps casalis* fed on J2 juveniles of *T. semipenetrans*, J2 juveniles of *M. incognita*, and egg mass of *M. incognita* at 30 ± 1 °C, 55 ± 5% RH.

Prey Species	*Androlaelaps casalis*	
	Total Fecundity ± SD	Daily Fecundity
J2 juveniles of *T. semipenetrans* *n* = 40	48.72 ± 1.36 a	1.37 ± 0.06
J2 juveniles of *M. incognita* *n* = 40	46.50 ± 2.07 a	1.36 ± 0.09
Egg mass of *M. incognita* *n* = 40	12.45 ± 1.90 b	0.69 ± 0.14

Values followed by different letters within the same column are significantly different according to Tukey’s test at the 5% significance level.

**Table 5:** Predation rate of *Androlaelaps casalis* fed on J2 juveniles of *T. semipenetrans*, J2 juveniles of *M. incognita*, and egg mass of *M. incognita* at 30 ± 1 °C, 55 ± 5% RH.

Predator	Predator Gender	No. Consumed					
		J2 Juveniles of *T. semipenetrans* *n* = 40	Daily Rate	J2 Juveniles of *M. incognita* *n* = 40	Daily Rate	Egg Mass of *M. incognita* *n* = 40	Daily Rate
		Total Average		Total Average		Total Average	
Larva	Female Male	49.25 ± 2.22 40.45 ± 3.11	49.25 40.45	46.33 ± 1.40 42.64 ± 1.25	46.33 42.64	2.00 ± 0.04 2.00 ± 0.06	1.48 1.60
Protonymph	Female Male	82.80 ± 3.63 77.20 ± 2.55	75.27 73.52	72.15 ± 2.85 64.38 ± 2.87	68.71 64.38	5.75 ± 0.8 4.50 ± 0.11	2.35 1.95
Deutonymph	Female Male	129.14 ± 4.38 105.30 ± 3.87	107.61 105.30	135.77 ± 3.19 122.28 ± 3.00	96.97 90.57	7.75 ± 0.10 6.50 ± 0.12	3.10 2.70
Total	Female Male	261.19 ± 5.18 a 222.95 ± 4.75 a	79.14 73.09	254.25 ± 5.09 a 229.30 ± 4.56 a	73.69 68.44	15.50 ± 0.32 b 13.00 ± 0.28 b	2.48 2.18
Pre-oviposition	Female	114.87 ± 4.24	89.74	111.45 ± 5.22	82.55	12.90 ± 0.20	3.70
Oviposition	Female	3897.66 ± 12.85 a	109.83	3315.74 ± 8.30 b	97.09	80.41 ± 1.35 c	4.49
Post-oviposition	Female	158.37 ± 3.17	33.27	145.68 ± 3.62	29.43	9.80 ± 0.25	1.09
Longevity	Female Male	4170.90 ± 16.74 a 3146.85 ± 9.20 a	105.96 83.91	3572.87 ± 13.78 b 2795.28 ± 11.57 b	93.40 76.06	103.11 ± 1.18 c 76.28 ± 1.39 c	3.40 2.76
Life span	Female Male	4432.09 ± 16.64 a 3369.80 ± 13.71 a	98.88 79.10	3827.12 ± 18.21 b 3024.58 ± 15.82 b	87.17 71.84	118.61 ± 2.25 c 89.28 ± 1.89 c	3.24 2.65

Different letters in horizontal columns denote significant differences (F-test, *p* < 0.01).

**Table 6:** Effect of different prey species on the life table parameters of *Androlaelaps casalis* fed on J2 juveniles of *T. semipenetrans*, J2 juveniles of *M. incognita*, and egg mass of *M. incognita* at 30 ± 1 °C, 55 ± 5% RH.

Parameters	Prey Species		
	J2 Juveniles of *T. semipenetrans* *n* = 40	J2 Juveniles of *M. incognita* *n* = 40	Egg Mass of *M. incognita* *n* = 40
Net reproduction rate (*R_o_*)	30.19 ± 2.14	28.49 ± 2.21	8.79 ± 1.24
Mean generation time (*T*) (days)	24.37 ± 0.68	23.96 ± 0.72	31.52 ± 2.04
Intrinsic rate of increase (*r_m_*) day^−1^	0.286 ± 0.02	0.279 ± 0.02	0.092 ± 0.01
Finite rate of increase (λ) day^−1^	1.522 ± 0.01	1.498 ± 0.02	0.617 ± 0.01

## Data Availability

Data are contained within the article.
